# Stress and Anxiety Scores in First and Repeat IVF Cycles: A Pilot Study

**DOI:** 10.1371/journal.pone.0063743

**Published:** 2013-05-23

**Authors:** Kathy Turner, Margaret F. Reynolds-May, Emily M. Zitek, Rebecca L. Tisdale, Allison B. Carlisle, Lynn M. Westphal

**Affiliations:** 1 Department of Obstetrics and Gynecology, Stanford University School of Medicine, Stanford, California, United States of America; 2 Department of Psychiatry, Stanford University School of Medicine, Stanford, California, United States of America; 3 Department of Psychology, Stanford University, Stanford, California, United States of America; Tehran University of Medical Sciences, Islamic Republic of Iran

## Abstract

**Background:**

The role of stress in reproduction, particularly during treatment for infertility, has been of considerable interest; however, few studies have objectively measured stress and anxiety over the course of the IVF cycle or compared the experience of first-time and repeat patients.

**Methods:**

This prospective cohort pilot study enrolled 44 women undergoing IVF at a university-based clinic to complete the State-Trait Anxiety Inventory (STAI), Perceived Stress Scale (PSS) and Infertility Self-Efficacy Scale (ISES) at three time points prior to ovarian stimulation (T1), one day prior to oocyte retrieval (T2), and 5–7 days post embryo transfer (T3).

**Results:**

Mean STAI State scores were significantly elevated at all three time points (p<0.01). STAI State and PSS mean values did not change over time and did not differ in first-time vs. repeat patients. Self-efficacy (ISES) scores declined over time, with a greater decline for repeat patients. Of the 36 women who completed a cycle, 15 achieved clinical pregnancy. Using logistic regression modeling, all scores at T2 were correlated with pregnancy outcome with lower scores on the STAI State and PSS and higher scores on the ISES associated with higher pregnancy rates.

**Conclusions:**

Stress and anxiety levels remained elevated across all cycles. Women with lower stress and anxiety levels on the day prior to oocyte retrieval had a higher pregnancy rate. These results emphasize the need to investigate stress reduction modalities throughout the IVF cycle.

## Introduction

The role of stress in reproduction, particularly in relation to assisted reproductive technologies such as IVF, has long been a topic of interest. Questions include whether the process of fertility treatment is stressful, whether stress or anxiety has an impact on success of fertility treatment, and whether interventions to decrease stress are useful. The utility in discovering the answers lies not only in strengthening the efficacy of our fertility treatment approaches, but also in understanding the experience of infertility and its treatment as a quality of life issue.

Stress has proven difficult to define in standardized, measurable terms; in fact, the concept of stress remains subject to varied interpretations in the literature to date [1]. Reflecting this, a number of tested instruments have been developed to assess and measure stress, in particular relating stress to concepts of anxiety, self-efficacy, and coping, among others. Of these, the Spielberger State-Trait Anxiety Inventory (STAI) [2] is the most commonly used validated self-report assessment tool for the experience of anxiety as it is related to stress, differentiating between the situational experience and the overarching predisposition toward stress or anxiety with the “State” and “Trait” subscales.

While it has long been assumed that the condition of infertility is inherently stress-invoking, studies to date have been equivocal with some showing higher STAI scores compared to norm groups and others not (see [3], [4], [5] for reviews). Stress during fertility treatment is thought to be multidimensional; in addition to any psychological stress related to the diagnosis of infertility, there are potential stresses related to the medical procedures, the awaiting of a positive outcome, and the physiological effects of gonadotropin stimulation. A number of studies have looked at stress over the course of the IVF cycle [6]–[15]; however, the time points examined and instruments used varied, and analysis of change over time was not uniformly performed. Only one of these studies controlled for first versus subsequent IVF cycles, without finding significant differences in STAI scores [7].

Evidence supporting the hypothesis that stress is negatively correlated with success of fertility treatment, as measured by pregnancy or live birth rates, has also been mixed, with some studies showing an association [6], [14], [16]–[18] and others finding no difference [10], [12], [13], [19]–[21]. Similarly, it remains unclear whether interventions aimed at stress reduction could impact these outcomes.

The aim of this pilot study was to describe stress and anxiety levels over three time points during the IVF cycle**,** with an interest in documenting the general pattern of stress across the treatment cycle, rather than stress related to a specific procedure. Additional goals were to compare the experience of first- time and repeat IVF patients, to assess the utility of three instruments: STAI, Perceived Stress Scale (PSS) and Infertility Self-Efficacy Scale (ISES) in characterizing stress and resilience in this population, and to determine the feasibility of our study design regarding recruitment and compliance as a first step in designing an intervention study to address stress reduction in IVF patients.

## Materials and Methods

### Participants

Women undergoing an initial or repeat IVF cycle at our university center from June 2009 to September 2009 were approached to participate in this study. Patients over 42 years of age, with an FSH level greater than 14 IU, or using frozen or donor embryos were excluded. Every eligible patient was identified through the clinic schedule and at weekly IVF clinic meetings; patients were then recruited at scheduled clinic visits or by phone or email prior to their clinic visit. Using this method, 90% of all new and returning IVF patients were successfully contacted.

**Figure 1:**
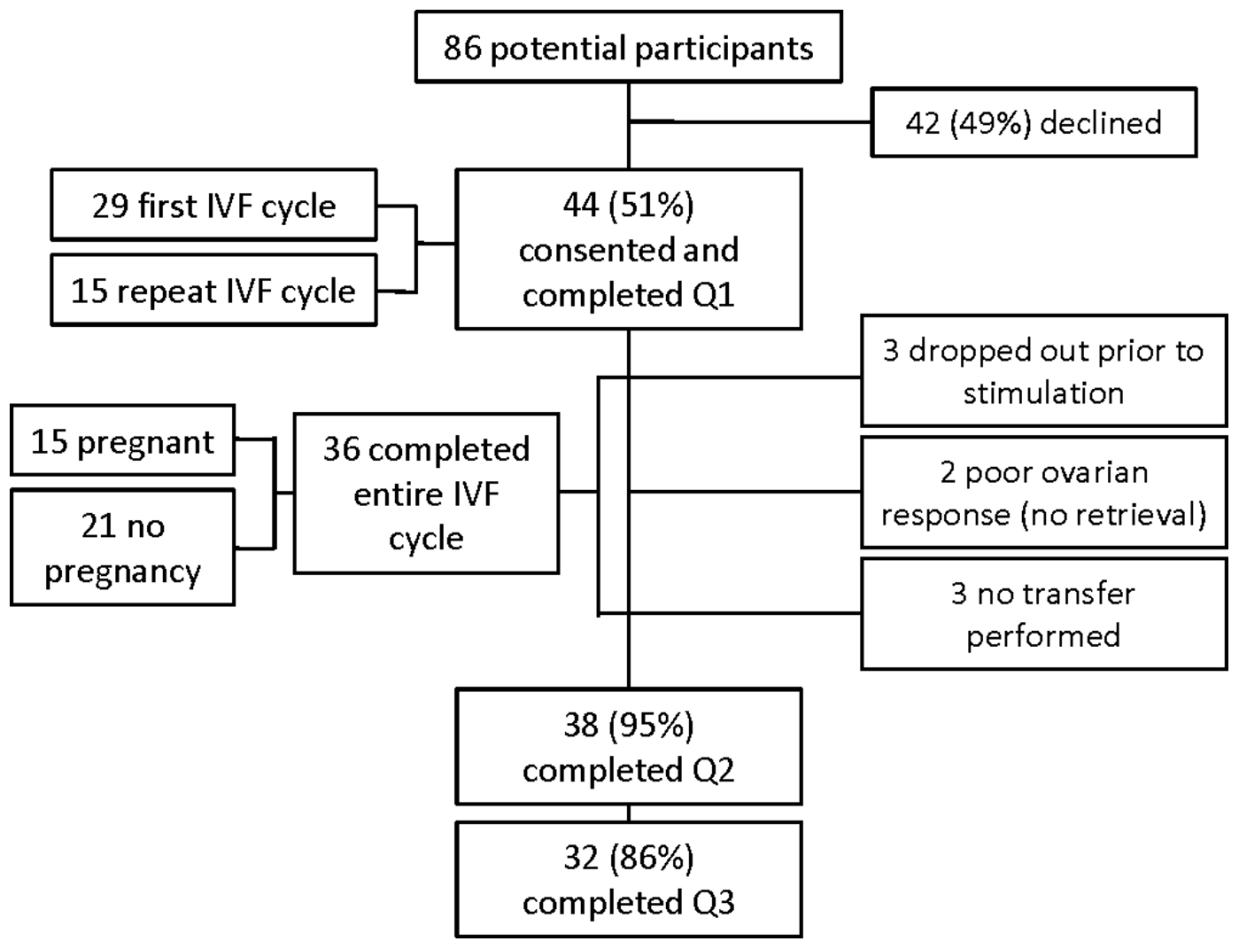
Participant completion.

A total of 86 women were approached, 44 of whom gave written consent to participate and completed baseline questionnaires (Figure 1) for a recruitment rate of 51%. Twenty-nine were entering their first IVF cycle; 15 were entering a repeat cycle. Three women dropped out prior to beginning stimulation because of work schedule conflict, need to treat male partner first, and pregnancy. Two women had cycles cancelled prior to oocyte retrieval because of poor ovarian response. Three women had no embryo transfer because of no viable embryos or ovarian hyperstimulation. A total of 36 women completed the entire cycle (82%). Of the 44 women enrolled, 38 completed T2 questions and 32 completed T3 questionnaires for an overall compliance rate of 86%. 5 out of the 36 patients with completed cycles failed to complete all three assessments. Twenty-seven participants (61%) used an antagonist protocol, 9 (21%) a long agonist protocol, and 8 (18%) a flare protocol.

### Materials

Participants filled out a series of three validated questionnaires: Anxiety was measured using the Spielberger State-Trait Anxiety Inventory (STAI), which consists of two subscales. State Anxiety (STAI-S) is a measure of situational anxiety with subjects being asked to respond based on “how you feel right now”. Trait Anxiety (STAI-T) is a measure of a general tendency to be anxious with subjects being asked to respond based on “how you generally feel” [2]. Each subscale consists of 20 items scored on a four- point Likert-type scale; thus the range of possible scores on each subscale is 20 (low anxiety) to 80 (high anxiety). The reliability coefficient (Cronbach’s α), referring to a normative sample of men and women, is 0.91 for STAI-S and 0.90 for STAI-T. The test-retest reliability ranges from 0.92 (after 90 min) to 0.75 (after 118 days) for the STAI-T.

In published reports, average STAI scores in women undergoing fertility treatment range from 33 to 50 [4], [5], [7], [11], [12], [20]–[33]. According to the population samples published in the reliability and validity testing for the STAI in 1988, the average score among women was 35.2 [2]; however population norms have not been assessed in all countries where research on stress during IVF has taken place. Cut-off scores for the STAI demarcating low- or high-stress states (or traits) have not been adequately identified in the literature, particularly in this population.

Perception of stress was measured using the Perceived Stress Scale (PSS) 10-item version, which consists of 10 questions graded on a five-point Likert scale. Scores can range from 0 to 40, with higher scores indicating greater subjective distress. The PSS is designed to measure the degree to which individuals perceive their lives to be unpredictable, uncontrollable and overloaded [34]. Examples of questions include “In the past week, how often have you felt difficulties were piling up so high that you could not overcome them?” The normative sample mean for females in the general population is 13.7+/−6.6 [34]. In a study of fertile women attempting conception, the mean reported PSS scores ranged from 14.8–17.8 [35]. The PSS was first applied to an infertility-specific population in 2000 [36] and has subsequently been used in this population in a number of studies [22], [35], [37]–[39]. Cronbach’s α scores range from 0.67 to 0.86 in the 1988 validity study [34].

We selected the Infertility Self-Efficacy Scale (ISES) because its primary measure focuses on the perception of “positive” experiences and attributes in contrast to the STAI and PSS, which focus on “negative” elements of distress. The ISES attempts to measure resilience and an individual’s self-confidence in coping with the infertility diagnosis and treatment [37]. The questionnaire consists of 16 statements ranked on a 9-point Likert scale; higher scores indicate a greater degree of self-efficacy. Examples of ranked statements include “I feel confident I can make meaning out of my infertility experience,” with 1 representing “not at all confident” and 9 representing “totally confident.” The ISES was designed to measure an individual’s self-confidence in coping with infertility diagnosis and treatment. The scale was first published in 2006 [37] and has subsequently been used in a number of studies [40]–[43]. No population norm has been established. A published report of a stress reduction intervention in infertility patients found a baseline ISES mean of 73.5+/−22.1 [29]. The Cronbach’s α was reported to be 0.94 with a test- retest reliability (within one week) of 0.91 in the 2006 validity study [37].

### Methods

Written informed consent was obtained from all participants. This study was approved by the Stanford University Institutional Review Board. Participants filled out each of the three questionnaires at each of three time points: baseline before the start of ovarian stimulation (T1); 1 day prior to oocyte retrieval (T2); and 5–7 days post embryo transfer while awaiting a pregnancy result (T3). The selected times were designed to avoid the potential added stressors that may occur on the day of a medical procedure.

Since the three time points did not necessarily reflect participant points of contact with the clinic, questionnaire packets were mailed to all participants with instructions as to when each set needed to be filled out and mailed back. In addition, all participants were contacted by the study coordinator via phone or email the day before the questionnaires were due. Participants who completed questionnaires at all three time points received a gift card.

### Statistical Analysis

Independent samples t tests and chi-square tests were used to compare the demographic information of the first-time and repeat patients. One-sample t tests were used to compare mean STAI values to population means for women provided by the STAI Manual [2]. STAI State, PSS, and ISES scores were compared across the time points using mixed-models analysis in SAS version 9.1. In the mixed models analysis, we included a random intercept and the effects of time (categorical), patient status (first-time and repeat), and the time by patient status interaction. Twelve logistic regression models were used to predict pregnancy from each of the measures of stress, anxiety, and resiliency. Lastly, Pearson correlation scores were assessed between STAI, PSS, and ISES scales at each time point. Critical values were set at 5%.

## Results

Study population demographic and clinical characteristics as means ± SD are shown in Table 1. Demographics did not differ between first-time and repeat IVF patients except for FSH, which was higher in the repeat patients and number of embryos transferred, which was higher in the repeat patients with a p-value approaching significance.

**Table 1:** Demographic and clinical characteristics of all participants.

	Overall (n = 44)	First cycle (n = 29)	Repeat Cycle (n = 15)	p value
Age (years)	35.3±3.82	34.9±4.01	36.0±3.42	0.37
Race				0.08
Caucasian	29	17	12	
Asian	14	12	2	
African American	1	0	1	
BMI (kg/m^2^)	23.4±3.90	23.9±4.33	22.3±2.72	0.20
FSH (IU/L)	7.03±2.45	6.46±2.39	8.09±2.27	0.04
Number of Follicles	11.0±6.67	10.7±7.06	11.6±6.02	0.67
Number of Oocytes	10.4±7.56	10.1±7.79	10.9±7.30	0.74
Number of Embryos Transferred	2.05±1.48	1.76±1.18	2.60±1.84	0.07
Positive Pregnancy	15 (42%)^a^	9 (39%)^b^	6 (46%)^c^	0.68

*Note:* Values are means ± SD.

a Out of 36 patients who completed the cycle.

b Out of 23 patients who completed the cycle.

c Out of 13 patients who completed the cycle.

Table 2 shows mean values ± SD for the STAI (State and Trait), the PSS, and ISES for all participants at T1, T2, and T3 as well as broken down by first-time and repeat patients. Mean STAI-State scores were significantly elevated over the normative population mean of 35.20 at all three time points (all p values <0.01), and mean STAI-Trait scores were significantly elevated over the normative population mean of 34.79 at T1 (p = 0.02) and T2 (p = 0.02) but not at T3 (p = 0.14).

**Table 2:** T1, T2, and T3 scores in all participants.

	T1 (n = 44)	T2 (n = 38)	T3 (n = 32)	p value
**STAI State**	41.45±13.09	41.63±13.69	42.06±13.83	0.7
First-time	40.41±11.46	41.42±12.41	39.40±12.12	
Repeat	43.47±16.04	42.00±16.15	46.5±15.84	
**STAI Trait**	38.68±10.65	38.87±10.70	37.81±11.16	0.93
First-time	38.62±9.13	38.79±10.01	37.70±10.55	
Repeat	38.80±13.47	39.00±12.18	38.80±12.59	
**PSS**	14.61±6.49	14.84±6.50	15.56±6.67	0.18
First-time	14.72±4.60	13.96±5.22	14.60±5.65	
Repeat	14.40±9.33	16.36±8.26	17.17±8.11	
**ISES**	96.15^a^ ±24.31	92.05±26.61	89.28±27.01	0.01
First-time	99.11±18.88	94.04±24.50	95.45±24.53	
Repeat	90.00±32.89	88.64±30.56	79.00±28.84	

*Note:* Values are means ± SD.

*Note:* P values represent the main effect of time in the mixed models analysis.

a n = 43.

Mixed-model analyses were run to examine how the stress measures changed over time for first-time and repeat IVF patients. For the STAI State, STAI Trait, and PSS values, there was no main effect of time, no main effect of patient status, and no interaction between time and patient status. For the ISES scores, there was not a main effect of patient status (p = 0.27), but there was a main effect of time (p = 0.01) such that ISES scores significantly declined over the three time points overall. Furthermore, there was a significant interaction between patient status and time, such that the ISES scores decreased more for the repeat patients than for the first time patients (p = 0.045).

STAI State and Trait, PSS, and ISES scores at each time point were significantly correlated with each other (Pearson correlation scores ranging from 0.346 to 0.863, p values ranging from 0.053 to <0.0002). Age and BMI were not correlated with any anxiety or stress scores, however FSH was correlated with ISES at T3 (r = −0.36, p = 0.04) and number of follicles was correlated with STAI State at T2 (r = −.32, p = .047), STAI Trait at T1 (r = −.30, p = .049), and ISES at T2 (r = .35, p = .03). However, number of follicles, oocytes, or transferred embryos did not significantly differ between women who achieved pregnancy versus those who did not (p-values ranging 0.44 to 0.78, data not shown).

Of the 36 women who completed a cycle, 15 participants achieved a clinical pregnancy with documentation of fetal cardiac activity at gestational age 6–7 weeks. Demographics and clinical characteristics did not differ between these two groups. Table 3 shows stress and anxiety scores in women who achieved pregnancy versus those who did not. Using logistic regression models to predict pregnancy, we found that all scores at T2 were a significant predictor of pregnancy. Women with lower scores on the STAI and PSS, and higher scores on ISES, one day prior to oocyte retrieval were more likely to get pregnant (Table 3). When controlling for number of follicles, the models remained significant except for the STAI State model, which approached significance with a p value of 0.06 (data not shown).

**Table 3:** Logistic regression models predicting pregnancy at T1, T2, and T3.

		T1 (n = 36)	T2 (n = 35)	T3 (n = 32)
STAI State	Pregnant	37.53±12.33	34.93±11.18	41.36±14.20
	Not pregnant	43.57±14.44	44.35±13.63	42.61±13.92
	p value	0.20	0.05	0.80
STAI Trait	Pregnant	35.93±11.00	33.67±8.10	34.29±9.55
	Not pregnant	38.86±10.88	41.30±10.34	40.56±11.80
	p value	0.42	0.03	0.12
PSS	Pregnant	12.67±7.12	11.53±6.49	16.14±7.09
	Not pregnant	15.43±6.12	16.20±5.09	15.11±6.50
	p value	0.22	0.03	0.66
ISES	Pregnant	103.16±25.00	106.73±17.38	94.21±25.28
	Not pregnant	91.33±22.35	84.95±27.13	85.44±28.39
	p value	0.16	0.02	0.36

*Note:* Values are means ± SD.

## Discussion

In this pilot study, we assessed anxiety, stress and resilience, as measured by the STAI, PSS and ISES questionnaires, respectively, at three time points in the IVF cycle. We found that anxiety and stress did not significantly change across the cycle, while resilience decreased over time, particularly for the repeat IVF patients. These findings add to a growing literature looking at stress over the IVF cycle. Although previous studies have found mixed results–with some finding no change in stress [9], [10], [14] and others finding significant change over the IVF cycle [7], [11], [13]–they are difficult to compare given varying methodology, including obtaining questionnaires at time points that may reflect procedure-related stress or anxiety. The time points selected for this study reflect our intention to identify and address anxiety as a quality of life issue throughout the IVF cycle rather than focusing on procedure-related anxiety. For this reason, the three time points specifically avoided the days of oocyte retrieval and embryo transfer.

In published reports, average STAI scores in women undergoing fertility treatment range from 33 to 50 [5], reflecting in part the variation in mean scores among different countries as well as differences in population norms. In our study, STAI-State mean scores were significantly elevated over the normative population mean of 35.20 at all three time points, thus supporting the need for stress reduction in this population.

The capacity to determine whether one of the three time points was more stressful than others could help guide the direction of future intervention studies. We found that stress and anxiety did not change significantly over the three time points. However, all scores at Time 2 were predictive through logistic regression modeling of successful pregnancy (with lower scores on STAI-State and PSS and higher scores on the ISES associated with higher pregnancy rates), suggesting that stress reduction prior to oocyte retrieval should be a future area of study.

One proposed explanation for the predictive dimension of the Time 2 scores is that patients may be aware of the number of follicles visualized on ultrasound before filling out the Time 2 questionnaires prior to oocyte retrieval. If women have a large number of visualized follicles, they may feel less stressed or anxious prior to retrieval; at the same time, a higher number of follicles may also be a predictor of successful IVF outcome. Our analysis supported the idea that number of follicles is related to stress level at Time 2, though it was not systematically assessed whether patients were informed of this number routinely. When we controlled for number of follicles in our logistic regression model, the STAI Trait, PSS, and ISES models remained significant, while the STAI State model approached significance with a p-value of 0.06, suggesting that this explanation does not fully address the documented phenomenon.

Another goal of this study was to determine if there was a difference in IVF-related stress between first-time and repeat IVF patients. Though limited by our small population, we found no difference in anxiety and perceived stress in these subgroups across the three time points. However, repeat patients showed a significantly greater drop in resilience (ISES) across the cycle than first-time patients. It is possible this may reflect an effect of burn-out associated with the cumulative stresses of multiple cycles, but it is not clear why other measures of stress and anxiety are not also implicated; this remains to be explored in future studies.

In this pilot study, our observation was that anxiety, as measured by STAI State scores, appears to be more variable in this population than anticipated. Our original estimate of the standard deviation was 8.5; our findings show it to be 13.8. Based on these findings, it would require a sample size of 272 to detect a difference of five points on the STAI State score in new vs. return patients and a sample size of 307 to detect a difference of five points in pregnant vs. not pregnant patients.

A secondary objective of this pilot study was to assess methodological feasibility prior to designing an intervention study. In the absence of an accepted and convenient biomarker for stress, we used three previously developed and tested instruments for characterizing stress. We chose the STAI because it is a well-established measure of anxiety that has been used extensively in this population and is sensitive to short-term change. We wanted to determine if using the PSS to measure perceived stress would provide additional information about participants’ experience over time. In addition, we wanted to use an infertility-specific instrument. The ISES asks women to rate how confident they feel about their ability to handle some of the stressful events related to their infertility treatment, which we used as a measure of resilience. Scores on all three instruments were significantly correlated at each time point, as would be expected given that the questionnaires aim to measure similar concepts though each through a unique lens. Though the ISES shows promise as a measure of coping and resilience in this population, it remains to be explored in larger studies whether any of the questionnaires has superior utility.

Compliance remains an issue in studies such as this that require participants to complete multiple questionnaires at several time points, particularly when the time points do not coincide with an office visit. A strength of our study was a high compliance rate (86%). We found that sending and receiving questionnaires through the mail, along with a phone or email reminder, was reliable and effective. In addition, we found that a small payment, in the form of a $10 gift card that participants received after returning all questionnaires, worked well as an incentive. A large percentage of our IVF patient population is made up of professional women working full-time or living a significant distance from the clinic. This raises challenges in designing an intervention study with minimal impact on participant time and with treatment options that would be perceived as valuable.

Limitations of our study design include small sample size and the inherent constraints of self-report measures of stress and anxiety. Stress is a difficult concept to operationalize, as has been explored considerably (see [1] for a review) and is currently lacking any widely accepted biomarker with significant sensitivity or specificity. It is possible that there was a selection bias of participants with women experiencing more anxiety and stress choosing not to enroll; alternatively, it is possible that women experiencing greater stress opted to enroll as a means of addressing this experience. Other considerations include the fact that the study population consisted of women presenting to an infertility clinic in a relatively affluent, suburban geographical area. Mean BMI in these patients is, for example, significantly lower than many population averages. These observations should be considered when attempting to generalize the results of this study.

### Conclusions

Overall, this pilot study found elevated stress and anxiety levels in women presenting for both first and repeat IVF cycles, with levels remaining elevated across the cycle. Lower stress and anxiety levels on the day prior to oocyte retrieval were associated with pregnancy success. These results emphasize the need to investigate stress reduction modalities throughout the cycle in adequately powered studies.
